# Quantification of ethanol in plasma by electrochemical detection with an unmodified screen printed carbon electrode

**DOI:** 10.1038/srep23569

**Published:** 2016-03-23

**Authors:** Gang Tian, Xiao-Qing Zhang, Ming-Song Zhu, Zhong Zhang, Zheng-Hu Shi, Min Ding

**Affiliations:** 1 Key Laboratory of Clinical Laboratory Diagnostics (Ministry of Education of China), College of Laboratory Medicine, Chongqing Medical University, Chongqing, 400016, P. R. China; 2 Center of Evidence Identification, Chongqing Police Bureau, Chongqing, China

## Abstract

Simple, rapid and accurate detection of ethanol concentration in blood is very crucial in the diagnosis and management of potential acute ethanol intoxication patients. A novel electrochemical detection method was developed for the quantification of ethanol in human plasma with disposable unmodified screen-printed carbon electrode (SPCE) without sample preparation procedure. Ethanol was detected indirectly by the reaction product of ethanol dehydrogenase (ADH) and cofactor nicotinamide adenine dinucleotide (NAD^+^). Method validation indicated good quantitation precisions with intra-day and inter-day relative standard deviations of ≤9.4% and 8.0%, respectively. Ethanol concentration in plasma is linear ranging from 0.10 to 3.20 mg/mL, and the detection limit is 40.0 μg/mL (S/N > 3). The method shows satisfactory correlation with the reference method of headspace gas chromatography in twenty human plasma samples (correlation coefficient 0.9311). The proposed method could be applied to diagnose acute ethanol toxicity or ethanol-related death.

Ethyl-alcohol (ethanol) is a commonly used and abused psychoactive component. High concentrations of ethanol (>1.0 mg/mL) in blood will induce mental confusion and impairment of cognitive function^1^ or direct death (>3.0 mg/mL)^2^. Therefore, rapid and accurate determination of ethanol concentration in blood is very crucial for individuals with the initially altered mental status or suspected ethanol intoxication. Additionally, for forensic purposes, the analyses of blood ethanol are regularly used as evidence for investigation of driving impairments and traffic accidents^3^,^4^.

Currently, gas chromatography (GC)^5^,^6^ and GC coupled with mass spectrometry (GC-MS)^7^,^8^,^9^ are the most commonly used techniques for quantitative measurement of ethanol concentration in blood. However, these techniques require experienced operators and relatively expensive instruments. In recent years, interests are boosting in the development of electrochemical biosensors due to their inherent advantages such as less reagent-consumption and compatible with portable devices^10^. In alcohol dehydrogenase (ADH)-based electrochemical biosensors, ADH catalyzes ethanol and cofactor nicotinamide adenine dinucleotide (NAD^+^) to acetaldehyde and dihytronicotinamide adenine dinucleotide (NADH), respectively. NADH is subsequently oxidized to NAD^+^ at the surface of the electrode and generates current signals. Therefore, ethanol is indirectly detected by electrochemical analyzers. The electrochemical oxidation of NADH requires high overpotential, which may increase interference with the substances in the samples^11^. To decrease the overpotential of electro-oxidation, enzymes such as ADH, NAD^+^, electro-mediators and other novel materials were immobilized on the surface of electrodes. These modification strategies have been successfully used for the measurement of ethanol concentration in wines^12^ and beverages^13^. Additionally, two studies first evaluated the recovery of ethanol in blood matrix by ADH-based electrochemical biosensors with the use of modified screen-printed carbon electrode (SPCE)^14^,^15^. Nevertheless, few studies analyzed ethanol concentrations quantitatively in blood samples^16^. Furthermore, step-by-step modification procedures on electrode surface were technically complicated and time-consuming^17^. Modified procedures by different laboratories and persons may increase total deviations and decrease the accuracy of the results^18^.

Herein, we developed an ADH-based indirect electrochemical method for accurate quantification of ethanol concentrations in plasma with unmodified SPCE. A simple and effective dilution strategy eliminates the matrix effect in plasma and ensures adequate detection sensitivity. This method was successfully applied to determine ethanol concentration in plasma samples of twenty volunteers.

## Results and Discussion

### Selection of electrodes

The electrochemical oxidation of NADH is related to material of the working electrode. Therefore, the working electrode made of Au, Ag or carbon was used to detect NADH (4.0 mM) in phosphate buffer saline (PBS, 200 mM, pH 10.4). The differential pulse voltammetry (DPV) curves showed similar oxidation peak potential of NADH (0.37 ± 0.02 V) on both SPCE (TE100) and Ag screen printed electrode (SPE). Whereas, higher peak current of NADH was observed (approximately 20 times) on SPCE than that on Ag-SPE, and the peak current of NADH on Au-SPE was much lower. Hence, SPCE was the best candidate for the determination of ethanol.

### Optimization of enzymatic reaction conditions

**Figure 1:**
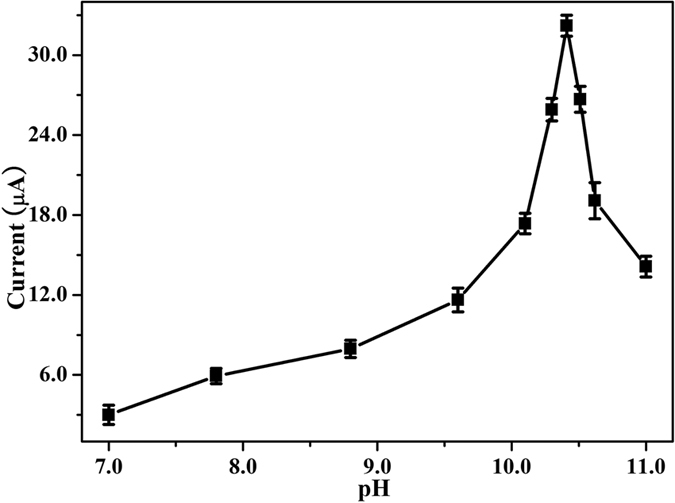
Effect of pH value on the current responses of ethanol solution (40.0 mg/mL, mean ± SD, n = 3).

Based on indirect electrochemical detection of ethanol, a series of enzymatic reaction conditions such as ionic strength of the supporting electrolyte, pH, concentrations of enzymes (NAD^+^, ADH), reaction time and temperature were taken into account in the experiment. PBS was selected as the supporting electrolyte with the concentration of 220 mM (Supplementary Fig. S1). The pH of PBS is a key factor which impacts not only on the enzymatic reaction but also on the electrochemical activity of bio-active substances. The effect of pH ranging from 7.0 to 11.0 was investigated in the manuscript (Fig. 1). The data showed that the peak current increased significantly with the increase of pH value, and reached a maximum value at pH 10.4, and then it decreased when pH is over 10.4. Consequently, the optimal pH of PBS was 10.4.

At the catalysis of ADH, ethanol reacts with excessive NAD^+^ to guarantee complete reaction. The effect of the concentrations of NAD^+^ (20 to 150 mM) and ADH (200 to 500 U/L) on the electrochemical responses were evaluated. The data illustrated that the optimal concentrations for NAD^+^ and ADH were 100 mM and 360 U/L, respectively (Supplementary Figs S2 and S3). Additionally, the reaction temperature and time were also investigated. In this study, the enzymatic reaction could be accomplished at room temperature within 12 min (Supplementary Figs S4 and S5).

### Matrix effects

**Figure 2:**
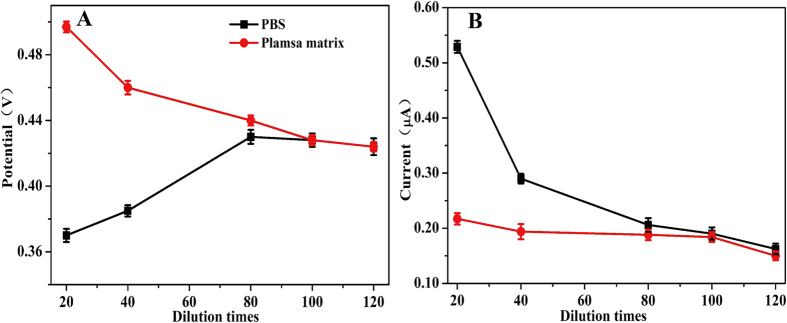
Matrix effects on the peak potential and peak current. (**A**) peak potential vs. dilution times, (**B**) peak current vs. dilution times. The spiked concentration of ethanol in plasma is 0.80 mg/mL (mean ± SD, n = 3).

Matrix effects, defined as the combined effect of all components of the sample other than the analyte on the measurement of the quantity, enable to suppress or enhance the response of analytes^19^. Dilution with solvent is a commonly proposed strategy to reduce or eliminate matrix effects^19^,^20^. To investigate the matrix effects of plasma, mixed blank plasma and PBS (220 mM, pH 10.4) were both spiked with ethanol (0.80 mg/mL), diluted with PBS (220 mM, pH 10.4) at 20, 40, 80, 100, and 120-fold, and measured quantitatively, respectively. As shown in Fig. 2, at 20-fold dilution, the peak potential in plasma matrix was higher than that in PBS (approximately 0.50 V vs. 0.37 V) (Fig. 2A), while the peak current was lower (about 2.20 μA vs. 5.40 μA) (Fig. 2B). Increasing the dilution from 40 to 100-fold, the peak potential decreased significantly, whereas the peak current was almost stable in plasma matrix. On the contrary, the peak potential was elevated slightly while the peak current decreased sharply in PBS. These results illustrated that matrix effects in plasma interfered with the oxidation of NADH at the surface of electrodes, which resulted in higher peak potential and lower peak current. 100 times of dilution was adopted in the subsequent work which was effective to eliminate matrix effects of plasma and guarantee the high sensitivity.

### Method validation

#### Calibration curve and sensitivity

Calibration curve of ethanol shows a linearity of the current X(nA) and concentration (Y) of ethanol in plasma ranging from 0.10 to 3.20 mg/mL with the limit of detection is 40.0 μg/mL (S/N > 3) in plasma. The average regression equation of the calibration curves is Y = 68.79ln(X) +164.06 (r = 0.9943, n = 5).

#### Specificity

**Figure 3:**
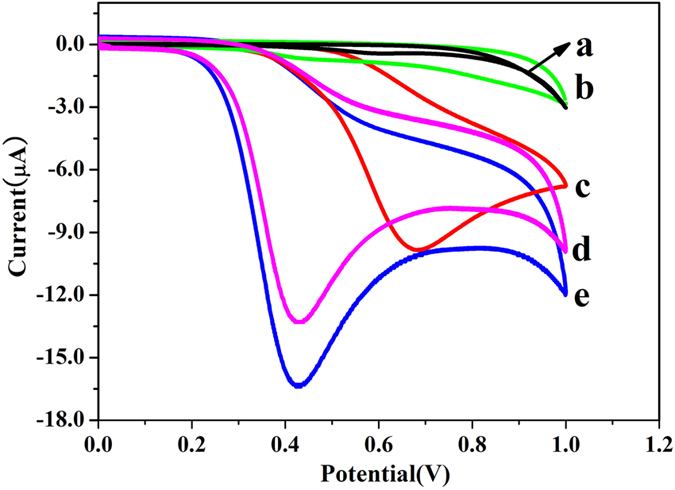
Cyclic voltammetry (CV) curves in 220 mM PBS (pH 10.4) (**a**), blank plasma in PBS (**b**), 4 μL NAD^+^ (100 mM), 4 μL ADH (360 U/L) and 4 μL spiked ethanol (20.0 mg/mL) plasma in 80 μL PBS (**c**), 4 μL NAD^+^ (100 mM), 4 μL ADH (360 U/L) and 4 μL 100-fold dilution spiked ethanol (20.0 mg/mL) plasma in 80 μL PBS (**d**), 0.05 mM NADH in PBS (**e**).

As given in Fig. 3, no oxidation peaks were observed in PBS (a) and blank plasma in PBS solution (b). The peak potential in spiked ethanol plasma in PBS solution was the highest at about 0.70 V (c). 100 times dilution of the spiked ethanol plasma with PBS, the peak potential decreased significantly (d), which was consistent with the spiked NADH in PBS (approximately, 0.42 V) (e). These results indicated that ethanol in plasma was indirectly measured by the electrochemical assay, and the matrix effect in plasma was negligible after 100-fold of dilution with PBS.

#### Precision and recovery

Precision was expressed by the relative standard deviation (RSD). As shown in Table 1, the validation of precision ranged from 7.2% to 9.4% for intra-day and 5.1% to 8.0% for inter-day, respectively. The mean recoveries of ethanol in plasma at low, medium and high concentrations (0.10, 0.40, 1.60 mg/mL) ranged from 80.1% to 100% for intra-day, and 81.9% to 103% for inter-day, respectively, achieving an acceptable recovery.

#### Interference of test

The concentration of ethanol in plasma before and after adding standards of hemoglobin or bilirubin was defined as *X*_C_ and *X*_T_, respectively. The interference value (expressed as X_T_ − X_C_) less than 1.96S showed insignificant interference and was expressed by *N*, while the interference value more than 1.96S indicated significant interference and was expressed by *I*. The interference value showed insignificant interference when the concentration of hemoglobin and bilirubin was less than 16.8 mg/mL and 200 μmol/L, respectively. Therefore, mild hemolytic or jaundice in plasma have no interference with the detection of ethanol (Table 2).

### Clinical application

**Figure 4:**
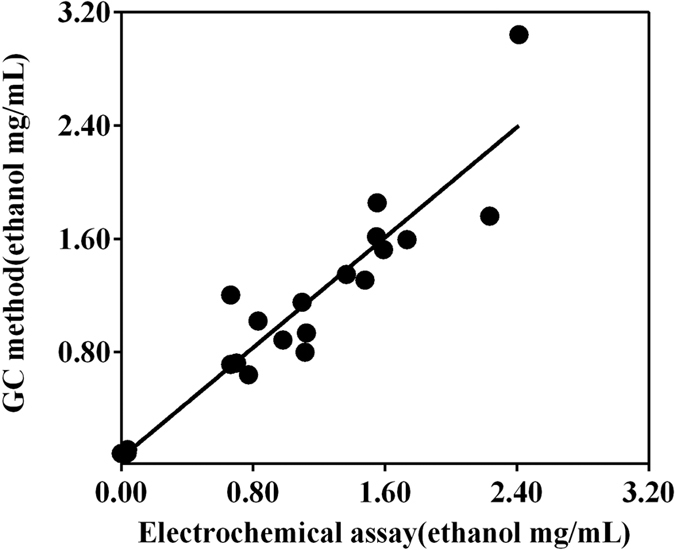
Correlation of ethanol concentrations in twenty plasma samples measured by the electrochemical assay and the reference method of gas chromatography (GC) method. The linear correlation is illustrated (r = 0.9311, p = 0.000).

Plasma samples of twenty volunteers were both analyzed by the electrochemical assay and the reference method of headspace GC. Pearson’s correlation analysis reveals a significant relationship of ethanol concentrations in plasma measured by both electrochemical method and the reference method (*r* = 0.9311, p = 0.000) (Fig. 4). The equivalent test was used to further evaluate the deviations between the methods. The cutoff value of equivalent (δ) was ±0.011 mg/mL to ensure less than 10% of RSD in the reference method. The data indicated that the electrochemical assay and the reference method were equivalent (t_1_ = −0.06, *P*_1_ = 0.52; t_2_ = 0.20, *P*_2_ = 0.42) at 95% confidence interval and nineteen degrees of freedom.

Three types of amperometric sensors have been reported to determinate ethanol with SPE^14^,^15^,^16^. The electrodes of different materials or catalytic strategies, linear range, detection limits, sensitivity and response times were compared, respectively (see supplementary Table 1). As shown in supplementary Table 1, the recovery was acceptable when compared with previous studies^14^,^15^. Additionally, this method exhibited similar linear range, LOD and sensitivity to measure the concentrations of ethanol in plasma with a previous study^16^.

In conclusion, we have developed a novel indirect electrochemical method for the quantification of ethanol in plasma with unmodified SPCE. We eliminated the matrix effects of plasma with direct dilution and avoided tedious modification process. The sensitivity of the method was sufficient, which covered fairly low and lethal ethanol concentrations in blood. The precision and recovery were acceptable. This method was applied to determine the concentrations of plasma ethanol in twenty volunteers. This encouraging work is to develop of a simple, accurate and practical electrochemical method for the determination of ethanol in plasma for clinical diagnosis or forensic purposes.

## Methods

### Materials and reagents

Oxidized nicotinamide adenine dinucleotide (NAD^+^, No: 10621650001) and alcohol dehydrogenase (ADH, 361U/mg) were purchased from Roche Company (Switzerland). Ethanol was purchased from Chongqing Chuandong Chemical Company (Chongqing, China). All other reagents were analytical grade (AR). Sodium phosphate dibasic (Na_2_HPO_4_) and sodium phosphate (Na_3_PO_4_) were obtained from Kelong Chemical Company (Chengdu, China). Ultrapure water was supplied from a Millipore water purification system (≥18 MΩ, Milli-Q, Millipore).

### Apparatus

All electrochemical experiments were performed with a CHI 852C type electrochemical analyzer (Shanghai Chenhua Instruments Co. Ltd., China). Screen-printed electrodes were purchased from Zensor Company (Taichung, Taiwan, WWW.Zensor.com.tw/). Three-electrode system consists of a working electrode (geometric area, 0.071 cm^2^), Ag/AgCl (3.0 M, KCl) reference electrode, and a platinum auxiliary electrode.

### Preparation of standard solutions and plasma samples

Standard stoke solutions of NAD^+^ (20 to 160 mM) and ADH (200 to 500 U/L) were prepared with ultrapure water and stored at −20 °C. Standard solution of ethanol was prepared freshly before use and diluted serially with ultrapure water to produce the working solution (1.0, 2.0, 4.0, 8.0, 16, 32 and 48 mg/mL).

Twenty volunteers were recruited from the Chongqing Medical University. Prior informed consent was obtained in writing from each volunteer included in the study. This study was approved by the local ethics committee of the Chongqing Medical University. All clinical investigations were conducted in accordance with the Declaration of Helsinki, and all methods were carried out in accordance with the approved guidelines. Venous blood of each volunteer was collected into two heparinized tubes after drinking amount of wine randomly. One blood sample was sent to the Material Evidence Identification Center of Chongqing (an institution for forensic blood alcohol analysis in Chongqing, China). The process of measurement was in according to the formal document (SF/Z JD0107001-2010, China). The other paired blood sample was immediately centrifuged at 3000 g for 10 min to separate plasma to detect ethanol by electrochemical method. All samples were sealed and stored at 4 °C, and the measurement were finished within one day.

### Validation of the electrochemical method

Ethanol calibration solutions were prepared by adding ethanol standard solution (100 μL) into mixed blank human plasma (900 μL) to generate a concentration of 0.10, 0.20, 0.40, 0.80, 1.60, 3.20 and 4.8 mg/mL, respectively. The linearity of the calibration curves (ranging from 0.1 to 3.2 mg/mL) was assessed based on six sets independently prepared calibrations curves using linear regression. Specificity was estimated to compare the peak potential in220 mM PBS (pH 10.4) (a), blank plasma in PBS (b), 4 μL NAD^+^ (100 mM), 4 μLADH (360 U/L) and 4 μL spiked ethanol (20.0 mg/mL) plasma in 80 μLPBS (c), 4 μL NAD^+^ (100 mM), 4 μLADH (360 U/L) and 4 μL100-fold dilution spiked ethanol (20.0 mg/mL) plasma in 80 μL PBS (d) and 0.05 mM NADH in PBS (e), respectively. Precision were evaluated by measuring five replicates at three concentrations levels of spiked ethanol (0.10, 0.40, 1.60 mg/mL) mixed plasma on the same day (inter-day) and on five consecutive days (intra-day). The recovery of ethanol was determined by measuring five replicates at three concentrations levels of spiked ethanol (0.10, 0.40, 1.60 mg/mL) mixed blank plasma. Data analysis was performed with SAS^®^ Deployment Wizard 9.3 (USA).

Interference of test was according to the guideline of the Clinical and Laboratory Standards Institute (CLSI) in clinical chemistry (CLSI document: EP07-A2, http://shop.clsi.org/method-evaluation-documents/EP07.html). It is necessary for the assessment of main interference such as hemoglobin and bilirubin in blood analysis. To evaluate the selectivity of this assay, different concentrations of hemoglobin (0 to 50 mg/mL) and bilirubin (0 to 400 μmol/L) were added into spiked ethanol (0.16 mg/mL) plasma, respectively.

### Electrochemical detection

Cyclic voltammetry experiments were performed at a scan rate of 100 mV s^−1^ with voltage from 0 to 1.0 V). The measurement of ethanol in plasma was performed by differential pulse voltammetry (DPV) measurement with parameters of voltage (0∼1.0 V), increment (0.005 V), amplitude (0.10 V), pulse width (0.05 s) and pulse period (0.2 s). 10 μL of plasma sample was diluted 100 times with PBS (pH 10.4, 220 mM). Then, 4 μL diluted plasma, 4 μL 100 mM of NAD^+^ and 4 μL 360 U/L of ADH solution were added in 80 μL of PBS (220 mM, pH 10.4) and mixed completely. After reaction for 12 min in a sealed eppendorf tube at room temperature, 40 μL the mixed solution was put to the working electrodes.

## Additional Information

**How to cite this article**: Tian, G. *et al.* Quantification of ethanol in plasma by electrochemical detection with an unmodified screen printed carbon electrode. *Sci. Rep.* **6**, 23569; doi: 10.1038/srep23569 (2016).

## Supplementary Material

Supplementary Information

## Figures and Tables

**Table 1:** The precisions and recoveries of ethanol detected by electrochemical method in human plasma (n = 5).

Added concentration (mg/mL)	Measured concentration (Mean ± SD, mg/mL)	Precision (RSD, %)	Recovery (%)
Intra-day			
0.10	0.088 ± 0.008	9.4	80.1
0.40	0.381 ± 0.003	7.4	98.5
1.60	1.62 ± 0.11	7.2	100
Inter-day			
0.10	0.083 ± 0.007	8.0	81.9
0.40	0.380 ± 0.003	6.5	99.5
1.60	1.56 ± 0.01	5.1	103

**Table 2:** The effect of hemoglobin and bilirubin on the determination of ethanol in plasma.

Added hemoglobin (mg/mL)	Concentration of ethanol(mg/mL)			Added bilirubin (μmol/L)	Concentration of ethanol(mg/mL)		
	measured	*X*_T_ − *X*_C_	1.96 *S*		measured	*X*_T_ − *X*_C_	1.96 *S*
0.0	0.16	−	0.008	0.0	0.16	−	0.008
4.7	0.17	0.002	*N*	50	0.16	−0.001	*N*
16.8	0.17	0.007	*N*	100	0.15	−0.004	*N*
20.7	0.18	0.011	*I*	200	0.15	−0.007	*N*
30.9	0.19	0.027	*I*	300	0.13	−0.025	*I*
50.0	0.22	0.054	*I*	400	0.10	−0.057	*I*

*X*_C_: The concentration of ethanol in plasma before adding standards of hemoglobin/bilirubin; *X*_T_: The concentration of ethanol in plasma after adding standards of hemoglobin/bilirubin. *N*: no significant interference. *I*: significant interference.
