# Supplementary material for: Quantification of ethanol in plasma by electrochemical detection with an unmodified screen printed carbon electrode
Source: Sci Rep. 2016 Mar 23;6:23569. doi: 10.1038/srep23569 (PMC4804280; doi:10.1038/srep23569)
Supplement: Supplementary Information [file srep23569-s1.pdf]

Quantification of ethanol in plasma by electrochemical detection with an unmodified screen printed carbon electrode

Authors:

Gang Tian[1], Xiao-Qing Zhang[1], Ming-Song Zhu[1], Zhong Zhang[2], Zheng-Hu Shi[1] & Min Ding[1]

Author affiliations:

[1]Key Laboratory of Clinical Laboratory Diagnostics, Ministry of Education, College of Laboratory Medicine, Chongqing Medical University, Chongqing, 400016, China.
[2]Center of Evidence Identification, Chongqing Police Bureau, Chongqing, China.

Corresponding author:

Min Ding, College of Laboratory Medicine, Chongqing Medical University, Chongqing, 400016, China. Phone: +023-6848-5240; Fax: +023-6848-5240; E-mail: dingmin@cqmu.edu.cn.

Supplementary information

Table S1. Comparison of the electrochemical method with other amperometric sensors for the measurement of ethanol by screen printed electrode (SPE).

Biosensors/reference*	Linearity range (mM)/matrix	LOD (μM)	Sensitivity (nA/mM)	RT(s)	Recovery (%) /matrix
MDB/GMCs/CS/SPCE[14]	0.5-15 /Tris-HCl	80	67.28	5	97.2-106 /blood
Nafion/MB/SPCE[15]	1-5 /PBS	11	126.8	30	96.5-104 /serum
GNPs/MWCNT/MB/Nafion/ SPCE[16]	0.2-25 /PBS-blood<br>2.0-250 /blood	50<br>500	121.4<br>12.14	< 15	Not mentioned
Unmodified SPCE/this work	2.2-70 /plasma	850	7.84	-	80.1-103 /plasma

*screen printed carbon electrode (SPCE); limit of detection (LOD); Response time (RT), meldola's blue (MDB); graphitized mesoporous carbons (GMCs); chitosan (CS); Meldola's Blue (MB); gold nanoparticles (GNPs); multi-wall carbon nanotubes (MWCNT).

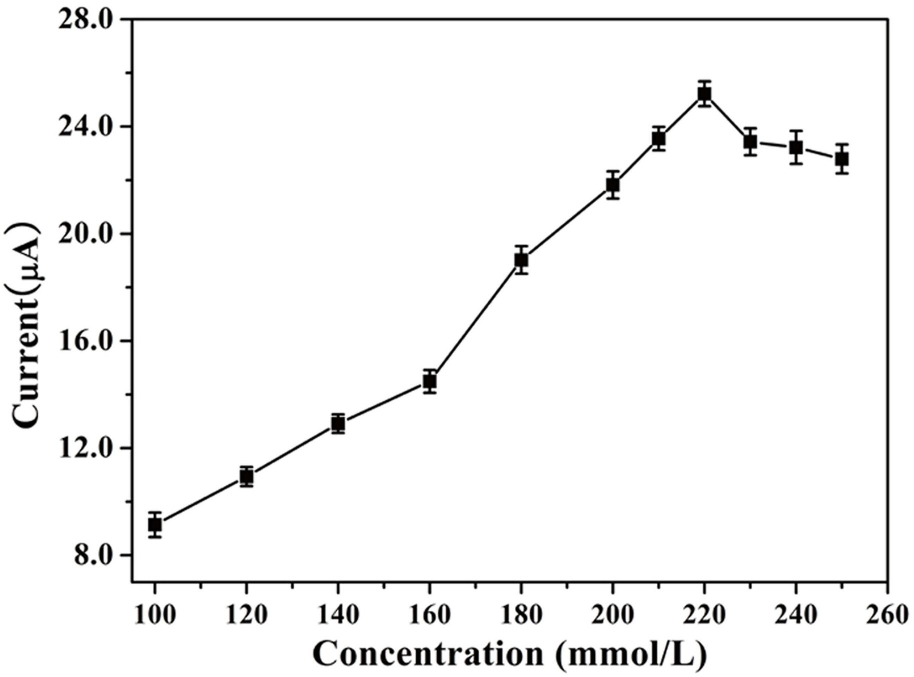

Figure S1. Effect of PBS concentrations on the current responses of ethanol solution (40.0 mg/mL, mean±SD, n=3).

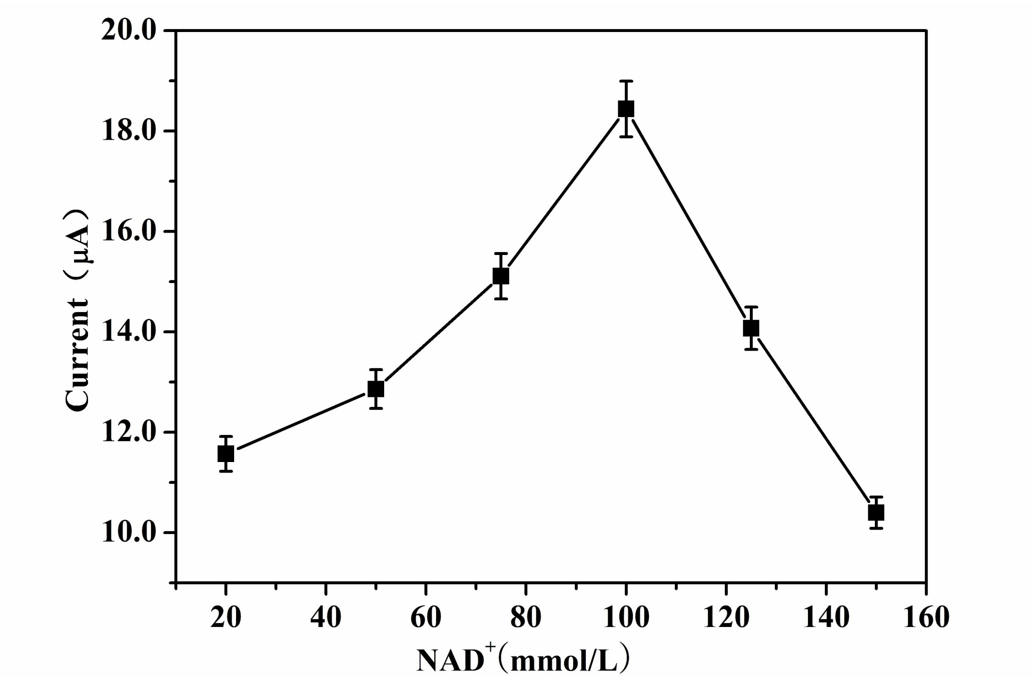

Figure S2. Effect of NAD^+ concentrations on the current responses of ethanol solution (40.0 mg/mL, mean±SD, n=3).

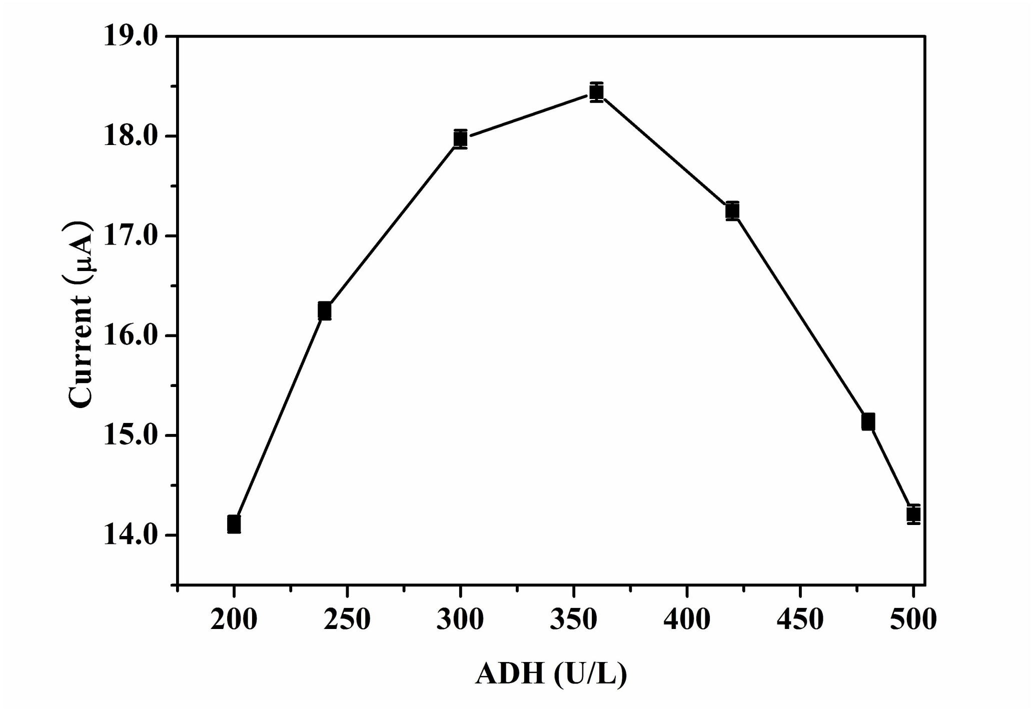

Figure S3. Effect of ADH concentrations on the current responses of ethanol solution (40.0 mg/mL, mean±SD, n=3).

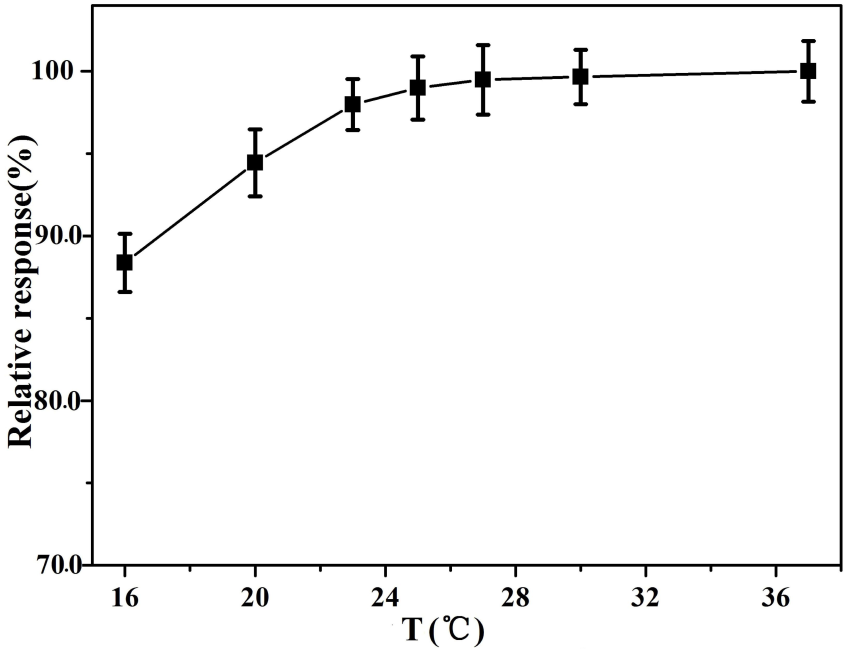

Figure S4. Effect of temperature (T) on the current responses of ethanol solution (40.0 mg/mL, mean±SD, n=3).

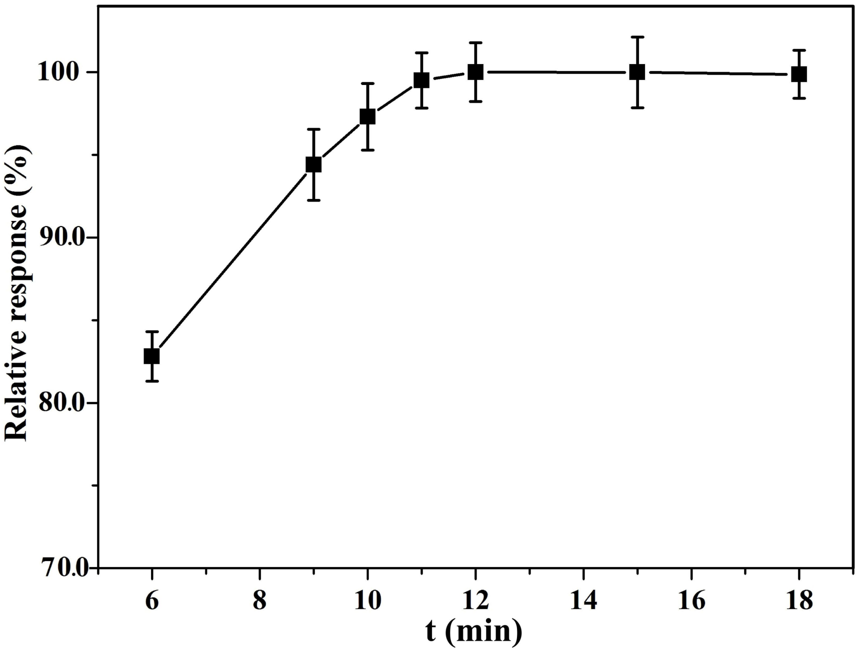

Figure S5. Effect of reaction time (t) on the current responses of ethanol solution (40.0 mg/mL, mean±SD, n=3).
